# Airport Noise and Self-Reported Sleep Insufficiency, United States, 2008 and 2009

**DOI:** 10.5888/pcd12.140551

**Published:** 2015-04-16

**Authors:** James B. Holt, Xingyou Zhang, Natalia Sizov, Janet B. Croft

**Affiliations:** Author Affiliations: Xingyou Zhang, Janet B. Croft, Centers for Disease Control and Prevention, Atlanta, Georgia; Natalia Sizov, Federal Aviation Administration, Washington, DC.

## Abstract

**Introduction:**

Sleep insufficiency is a major health risk factor. Exposure to environmental noise may affect sleep duration and quality. The objective of this study was to assess the relationship between airport noise exposure and insufficient sleep in the United States by using data from the Behavioral Risk Factor Surveillance System (BRFSS).

**Methods:**

Data on the number of days without enough rest or sleep for approximately 750,000 respondents to the 2008 and 2009 BRFSS were linked with data on noise exposure modeled using the US Federal Aviation Administration’s (FAA’s) Integrated Noise Model for 95 major US airports for corresponding years. Noise exposure data were stratified into 3 groups depending on noise levels. People living outside airport noise exposure zones were included as a reference category.

**Results:**

We found 8.6 mean days of insufficient sleep in the previous 30 days among 745,868 adults; 10.8% reported insufficient sleep for all 30 days; and 30.1% reported no days of insufficient sleep. After controlling for individual sociodemographics and ZIP Code-level socioeconomic status, we found no significant differences in sleep insufficiency between the 3 noise exposure zones and the zone outside.

**Conclusion:**

This research demonstrates the feasibility of conducting a national study of airport noise and sleep using an existing public health surveillance dataset and recommends methods for improving the accuracy of such studies; some of these recommendations were implemented in recent FAA-sponsored studies. Validation of BRFSS sleep measures and refined ways of collecting data are needed to determine the optimal measures of sleep for such a large-scale survey and to establish the relationship between airport noise and sleep.

## Introduction

Sleep is necessary for health and well-being (1). In *Healthy People 2020*, the US Department of Health and Human Services identified national health priorities and provided measurable objectives for health improvement goals, including sleep health: 1) increase the proportion of people who seek medical evaluation for obstructive sleep apnea; 2) reduce the rate of vehicular crashes caused by drowsy driving; 3) increase the proportion of students in grades 9 through 12 who get sufficient sleep; and 4) increase the proportion of adults who get sufficient sleep. The public health approach to these goals is multifaceted and involves addressing sleep environments (eg, living conditions, proximity to noise); the type, scheduling, and duration of work; associated health risk factors; chronic conditions, stress, and socioeconomic status; and validation of new and existing therapeutic technologies (2).

Healthy sleep is important for adults, infants, children, and adolescents. Adequate sleep is essential for childhood and adolescent development, is a major protective factor against infection, and supports metabolic functioning for diabetes prevention. It is necessary for performing well in school and for working safely and effectively. Sleep insufficiency is a major risk factor for adverse health outcomes, such as hypertension, heart disease, stroke, diabetes, depression, obesity, and injuries from motor vehicle crashes, industrial accidents, and medical and occupational errors, and mortality (1,3–6). Insufficient sleep can lead to patterns of behavior that may negatively affect family and interpersonal relationships and can result in productivity losses and limitations in daily functioning (7). *Healthy People 2020* Sleep Health Objective 4 is to increase the proportion of adults aged 22 years or older who sleep 7 or more hours during a 24-hour period (2).

Noise is commonly defined as unwanted sound. Roads, railroads, and airports are major sources of environmental noise at the population level. Environmental noise exposure is related to health risks and outcomes such as annoyance (8–10); work performance; increased aggression, depression, injuries, and accidents; and increased risk for hypertension and cardiovascular disease (11–18). Research on the association between environmental noise and sleep did not yield significant results or was potentially confounded by methodological factors (19–23).

Two ways in which environmental noise may affect health are 1) a direct pathway between noise and health or 2) an indirect pathway in which sleep is an intermediate factor. Studies on the effects of transportation noise on sleep generally take place in 1 of 2 settings — in a laboratory or in the field — and they are typically physiological studies on a small number of people. Some European data have been generated by combining data on national health cohorts and superimposed noise contours (13,14,24,25). Several local conditions besides noise might impair sleep, however, and often there is an assumption that such large national data sets are independent of local peculiarities. To answer the question about how airport noise affects sleep sufficiency, wide-scale epidemiological studies are needed. 

The objective of this study was to assess the independent effects of airport noise on sleep. We analyzed the association between exposure to airport noise and self-reported sleep insufficiency using data collected near 95 US airport locations during 2008 and 2009. This study is a first-of-a-kind attempt to investigate the relationship between airport noise exposure and self-reported insufficient sleep for the entire United States using public health surveillance data.

## Methods

We obtained data on individual health outcomes and sociodemographic characteristics from the 2008 and 2009 Behavioral Risk Factor Surveillance System (BRFSS) surveys. The BRFSS is a random-digit–dialed telephone survey of the noninstitutionalized US civilian population 18 years old or older. The survey is conducted in all 50 states, the District of Columbia, Guam, Puerto Rico, and the US Virgin Islands with collaboration from the Centers for Disease Control and Prevention (26). Of 847,116 completed interviews in 2008 and 2009, 745,868 (88%) respondents had a valid ZIP Code and answered the question “During the past 30 days, for about how many days have you felt you did not get enough rest or sleep?” We used these responses (365,326 from 2008 and 380,542 from 2009) for further analyses.

Sleep insufficiency is correlated with smoking status, weight, mental disorders, and insufficient rest, and the following populations are more likely to have insufficient rest or sleep: young adults, females, non-Hispanic blacks, those with low levels of education, and those who are unemployed or unable to work (1,27–29). To control for these variables, we included data on individual age (18–24 y and 25–34 y [combined for our analyses], 35–44 y, 45–54 y, 55–64 y and ≥65 y), sex, race/ethnicity (non-Hispanic white, non-Hispanic black, non-Hispanic other race, and Hispanic), and educational attainment (<high school graduate, high school graduate, some college, and ≥college graduate). We also included 2 known individual risk factors for insufficient sleep, smoking and obesity, to control for their effects on sleep. We categorized smoking status into current smokers, former smokers, and those who had never smoked. We used body mass index (BMI), calculated as self-reported weight in kilograms divided by the square of height in meters, to classify each respondent’s weight status. We categorized weight status into nonoverweight (BMI <25.0), overweight (BMI 25.0–29.9), and obese (BMI ≥30.0).

Data on ZIP Code population and median household income were obtained from Esri 2009 Demographics (Environmental Systems Research Institute, Inc). We categorized median household income into 4 groups on the basis of national quartiles (≤$37,349; $37,350–$45,526; $45,527–$57,748; and >$57,748).

Data on estimates of airport noise exposure for 2008 and 2009 were modeled by using the US Federal Aviation Administration’s (FAA’s) Integrated Noise Model (INM), which is widely used for environmental assessments. These area-level noise contours included 3 day–night average sound levels (DNLs) for each airport: 65 decibels (dB) or more, 60 dB to less than 65 dB, and 55 dB to less than 60 dB. DNLs computed in this study were based on fleet and number of operations information derived from Enhanced Traffic Management System (ETMS) data for the corresponding years and the same airport operational information used for National Airspace System annual assessment of performance. The 95 airports in our data set are the US airports for which noise data are available.

Five-digit ZIP Codes available in 2008 and 2009 were used to spatially link each BRFSS record to data on estimates of airport noise exposure and BRFSS outcomes in ArcGIS (Environmental Systems Research Institute, Inc). The estimates of airport noise exposure for individuals were thus based on the location of the ZIP Code’s geometric centroid in relation to the airport noise exposure zone (Figure). The distribution of selected characteristics, prevalence of sleep variables, and 95% confidence intervals (CIs) were obtained from weighted analyses. Multilevel logistic and linear regression models with county random effects were used to evaluate the effect of ZIP Code-level noise exposure on sleep sufficiency and to obtain β coefficients, odds ratios (ORs) and 95% CIs while controlling for individual sociodemographic characteristics (sex, age, race/ethnicity, education, smoking status, weight status, and survey year) and ZIP Code-level median household income. All analyses were performed in SAS (SAS Institute, Inc) and SUDAAN (Research Triangle Institute, Inc), which takes into account the complex survey design of the BRFSS.

**Figure:**
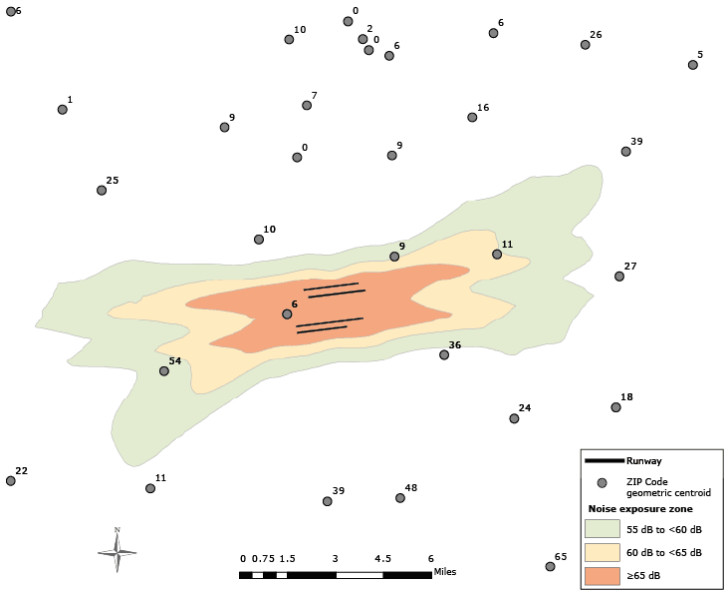
Example of airport noise exposure data and Behavioral Risk Factor Surveillance System (BRFSS) sample sizes at the ZIP Code level for 2008 and 2009. The contours represent 3 airport noise exposure zones. Next to each ZIP Code geometric centroid is the number of respondents to the BRFSS survey in that ZIP Code.

We treated the responses to the insufficient sleep question in 3 ways: 1) as a continuous variable (0–30 days), which is more intuitive for interpreting the impact of all relevant factors on sleep sufficiency; 2) as a dichotomous variable, with responses being either “all 30 days” or “fewer than 30 days”; and 3) as a dichotomous variable, with responses being either “0 days” or “at least 1 or more days.”

## Results

In 2008 and 2009, 855 (0.11%) of the 745,868 respondents lived in a ZIP Code in which the geometric centroid was in the airport noise exposure zone of 65 dB or more; 2,368 (0.32%) lived in the zone of 60 dB to less than 65 dB; 4,576 (0.61%) lived in the zone of 55 dB to less than 60 dB; and the remaining 738,069 (98.9%) lived outside these 3 zones.

We found no differences in the proportions of men, smokers, or obese people in any of the 3 exposure zones and people living outside these zones (Table 1). The 3 airport noise exposure zones had higher proportions of blacks and Hispanics, higher proportions of adults with less than high school education, and lower proportions of people aged 65 years or older compared with areas outside these zones (*P* < .05). The zone of more than 65 dB had a disproportionate share of ZIP Codes in which the median household income was significantly less likely to be in the highest quartile (>$57,748) and more likely to be in the second quartile ($37,350–$45,526) (*P* < .05) than it was in other zones.

**Table 1:** Distribution^a^ of Selected Characteristics of the Adult Study Population, by Airport Noise Exposure Zone, Behavioral Risk Factor Surveillance System, 2008 and 2009

Characteristic	Exposure Zone						Outside Exposure Zone (n = 738,069)	
	≥65 dB (n = 855)		60 dB to <65 dB (n = 2,368)		55 dB to <60 dB (n = 4,576)			
	n	% (95%CI)	n	% (95%CI)	n	% (95%CI)	n	% (95%CI)
**Year**								
2008	450	50.4 (43.4–57.3)	1,123	50.4 (46.2–54.5)	2,149	54.6 (51.5–57.7)	361,604	50.0 (49.8–50.2)
2009	405	49.6 (42.7–56.6)	1,245	49.6 (45.5–53.8)	2,427	45.4 (42.3–48.5)	376,465	50.0 (49.8–50.2)
**Sex**								
Male	306	50.2 (43.2–57.2)	865	49.6 (45.4–53.8)	1,682	48.5 (45.3–51.7)	287,314	49.7 (49.5–50.0)
Female	549	49.8 (42.8–56.8)	1,503	50.4 (46.2–54.6)	2,894	51.5 (48.3–54.7)	450,755	50.3 (50.0–50.0)
**Age, y**								
18–34	138	32.8 (25.5–41.0)	353	33.8 (29.5–38.3)	672	35.1 (31.6–38.7)	91,535	30.5 (30.2–30.8)
35–44	147	18.9 (14.5–24.2)	424	23.2 (19.8–27.0)	718	19.9 (17.6–22.5)	108,129	19.0 (18.8–19.2)
45–54	198	21.2 (16.5–26.9)	468	19.7 (16.7–23.0)	921	17.7 (15.7–19.8)	152,154	19.3 (19.1–19.4)
55–64	148	14.2 (10.6–18.7)	461	11.4 (9.5–13.7)	1,004	14.0 (12.5–15.7)	162,125	14.5 (14.4–14.6)
≥65	224	12.9 (10.1–16.3)	662	12.0 (10.4–13.8)	1,261	13.3 (11.9–14.8)	224,126	16.7 (16.5–16.8)
**Race/ethnicity**								
Non-Hispanic white	515	42.9 (36.5–49.5)	1,703	51.3 (47.1–55.5)	2,866	42.4 (39.5–45.4)	602,177	70.8 (70.5–71.0)
Non-Hispanic black	111	20.4 (15.0–27.1)	248	17.5 (14.3–21.3)	868	20.0 (17.6–22.6)	55,453	9.8 (9.6–10.0)
Hispanic	181	30.4 (23.4–38.4)	271	23.8 (19.8–28.4)	589	29.5 (26.4–32.8)	40,498	12.7 (12.5–12.9)
Non-Hispanic other	48^b^	6.4 (3.9–10.3)	146	7.4 (5.5–9.9)	253	8.1 (5.8–11.2)	39,941	6.7 (6.6–6.9)
**Education**								
<High school graduate	141	18.3 (12.5–26.1)	214	11.6 (8.9–15.0)	488	14.3 (12.3–16.6)	63,892	10.0 (9.8–10.2)
High school graduate	302	30.9 (25.2–37.3)	756	29.0 (25.5–32.8)	1,385	29.6 (26.7–32.6)	220,815	28.5 (28.2–28.7)
Some college	199	23.7 (18.3–30.0)	653	26.4 (22.6–30.4)	1,207	26.5 (23.4–29.8)	199,520	26.9 (26.7–27.1)
≥College graduate	213	27.1 (21.6–33.4)	745	33.0 (29.3–36.9)	1,496	29.7 (27.1–32.3)	253,842	34.6 (34.4–34.9)
**Smoking**								
Never smoked	480	62.1 (55.4–68.4)	1,146	55.0 (50.8–59.1)	2,347	60.6 (57.6–63.5)	389,236	56.5 (56.3–56.7)
Former smoker	204	17.8 (13.7–23.0)	734	22.8 (19.7–26.2)	1,337	20.6 (18.6–22.8)	224,033	25.0 (24.8–25.2)
Current smoker	171	20.1 (15.3–25.9)	488	22.2 (18.8–26.0)	892	18.8 (16.5–21.4)	124,800	18.5 (18.3–18.7)
**Weight status (kg/m^2^)**								
Nonoverweight (<25.0)	294	40.7 (33.6–48.3)	853	35.9 (32.1–39.9)	1,575	35.9 (32.7–39.3)	259,624	36.5 (36.2–36.7)
Overweight (25.0–29.9)	313	29.8 (24.7–35.5)	839	35.1 (31.2–39.2)	1,595	34.6 (31.8–37.5)	271,118	36.3 (36.1–36.6)
Obese (≥30.0)	248	29.5 (23.7–35.9)	676	29.0 (25.1–33.2)	1,406	29.5 (26.8–32.4)	207,327	27.2 (27.0–27.4)
**Household income^c^, $**								
≤37,349	89	13.6 (9.1–19.9)	391	23.7 (20.1–27.7)	904	21.8 (19.2–24.6)	131,085	13.3 (13.2–13.5)
37,350–45,526	422	43.4 (36.4–50.6)	353	11.0 (8.6–13.9)	636	17.8 (15.5–20.4)	158,625	17.8 (17.6–18.0)
45,527–57,748	167	23.7 (19.1–29.1)	821	26.9 (23.7–30.3)	1,147	23.1 (20.6–25.8)	197,853	25.6 (25.4–25.9)
>57,748	177	19.3 (14.8–24.7)	803	38.5 (34.4–42.7)	1,889	37.3 (34.3–40.4)	250,506	43.3 (43.0–43.5)

Abbreviations: CI, confidence interval; dB, decibel.

a Distribution percentages and 95% CIs obtained from weighted unadjusted analyses that take into account the complex sampling design.

b Estimate may be unstable when cell size <50 respondents.

c Household income based on medium household income of the ZIP Code.

The mean number of days of insufficient sleep in the previous 30 days (Table 2) was 8.6 (95% CI, 8.5–8.6 d) and was higher in 2008 than in 2009; higher among women than among men; lowest among those aged 65 or older than among those younger; highest among non-Hispanic blacks and lowest among Hispanics than among other racial/ethnic groups; highest among those with some college than among those with other education levels; highest among current smokers than former smokers or those who never smoked; highest among obese people than among overweight or nonoverweight people; and highest among those in ZIP Codes with the lowest median household income than among those in ZIP Codes with other income levels (all *P* values < .001). However, the mean number of days of insufficient sleep did not differ by airport noise exposure zone.

**Table 2:** Mean Number of Days and Prevalence of Insufficient Sleep or Rest in Previous 30 Days, by Selected Characteristics, Behavioral Risk Factor Surveillance System, 2008 and 2009

Characteristic	Sample, n	Number of Days, Mean (95% CI)	All 30 Days^a^, % (95% CI)	No Days^b^, % (95% CI)
**Total sample**	745,868	8.6 (8.5–8.6)	10.8 (10.7–11.0)	30.1 (29.9–30.4)
**Year**				
2008	365,326	8.7 (8.6–8.7)	11.1 (10.8–11.3)	29.9 (29.6–30.2)
2009	380,542	8.5 (8.4–8.5)	10.5 (10.3–10.7)	30.4 (30.1–30.7)
**Sex**				
Male	290,167	8.0 (7.9–8.1)	9.6 (9.4–9.9)	32.3 (32.0–32.7)
Female	455,701	9.1 (9.0–9.2)	11.9 (11.7–12.1)	28.0 (27.7–28.3)
**Age, y**				
18–34	92,698	10.1 (10–10.2)	12.6 (12.3–13.0)	21.1 (20.6–21.6)
35–44	109,418	9.8 (9.7–9.9)	11.9 (11.5–12.2)	21.4 (21.0–21.9)
45–54	153,741	9.0 (8.9–9.1)	11.0 (10.7–11.3)	26.0 (25.6–26.4)
55–64	163,738	7.4 (7.4–7.5)	9.7 (9.4–9.9)	36.4 (35.9–36.8)
≥65	226,273	4.8 (4.7–4.9)	6.9 (6.7–7.1)	56.2 (55.8–56.5)
**Race/ethnicity**				
Non-Hispanic white	607,261	8.6 (8.6–8.7)	10.5 (10.3–10.7)	29.6 (29.4–29.8)
Non-Hispanic black	56,680	9.1 (8.9–9.3)	13.4 (12.8–13.9)	29.2 (28.5–30.0)
Hispanic	41,539	7.9 (7.7–8.1)	10.1 (9.5–10.6)	32.6 (31.7–33.5)
Non-Hispanic other	40,388	8.5 (8.3–8.7)	11.5 (10.8–12.2)	32.3 (31.3–33.4)
**Education**				
<High school graduate	64,735	8.8 (8.6–8.9)	14.4 (13.8–15.0)	36.2 (35.3–37.0)
High school graduate	223,258	8.6 (8.5–8.7)	12.4 (12.1–12.7)	34.3 (33.8–34.7)
Some college	201,579	9.1 (9.0–9.2)	11.7 (11.4–12.0)	28.0 (27.6–28.4)
≥College graduate	256,296	8.0 (8.0–8.1)	7.7 (7.5–7.9)	26.6 (26.3–27.0)
**Smoking**				
Never smoked	393,209	8.1 (8.0–8.1)	8.9 (8.7–9.1)	28.9 (28.6–29.2)
Former smoker	226,308	7.9 (7.8–7.9)	10.2 (9.9–10.4)	35.9 (35.5–36.3)
Current smoker	126,351	11.0 (10.9–11.2)	17.4 (17.0–17.9)	26.1 (25.6–26.6)
**Weight status (kg/m^2^)**				
Nonoverweight (<25.0)	262,346	8.1 (8.1–8.2)	9.6 (9.4–9.9)	30.4 (30.0–30.8)
Overweight (25.0–29.9)	273,865	8.1 (8.0–8.2)	9.8 (9.6–10.1)	31.7 (31.4–32.1)
Obese (≥30.0)	209,657	9.7 (9.6–9.8)	13.6 (13.3–13.9)	27.8 (27.4–28.1)
**Household income^c^, $**				
<37,349	132,469	9.0 (8.9–9.1)	13.4 (12.9–13.8)	31.8 (31.2–32.4)
37,350–45,526	160,036	8.7 (8.6–8.8)	11.9 (11.5–12.3)	31.9 (31.4–32.4)
45,527–57,748	199,988	8.6 (8.5–8.7)	11.1 (10.8–11.4)	30.9 (30.4–31.3)
>57,748	253,375	8.3 (8.3–8.4)	9.4 (9.1–9.6)	28.5 (28.1–28.8)
**Noise zone**				
≥65 dB	855	8.2 (6.9–9.5)	9.8 (7.0–13.5)	32.1 (26.6–38.2)
60 dB to <65 dB	2,368	8.5 (7.7–9.3)	10.6 (8.5–13.1)	28.2 (24.6–32.0)
55 dB to <60 dB	4,576	8.1 (7.5–8.7)	9.7 (8.2–11.3)	31.0 (27.9–34.3)
Outside exposure zone	738,069	8.6 (8.5–8.6)	10.8 (10.7–11.0)	30.1 (29.9–30.4)

Abbreviations: CI, confidence interval; dB, decibel.

a Weighted unadjusted prevalence and 95% CIs of all 30 days versus zero to 29 days of reported insufficient sleep or rest in the previous 30 days.

b Weighted unadjusted prevalence and 95% CIs of no days versus 1 or more days of reported insufficient sleep or rest in the previous 30 days.

c Household income based on medium household income of the ZIP Code.

The percentage of respondents who indicated they had insufficient sleep for all of the previous 30 days (Table 2) was 10.8% (95% CI, 10.7%–11.0%), which was higher in 2008 than in 2009; higher among women than among men; highest among current smokers; and highest among obese people; the percentage declined with increasing age, increasing education, and increasing median household income (all *P* values < .001) (Table 2). However, the prevalence of sleep insufficiency for all 30 days did not differ by airport noise exposure zone.

The percentage of respondents who indicated they had insufficient sleep for none of the previous 30 days (Table 2) was 30.1% (95% CI, 29.9%–30.4%) and did not differ between survey year; it was higher among men than among women; higher among Hispanics than among non-Hispanic whites or blacks; and lowest among smokers (all *P* values < .001). The percentage increased with increasing age and declined with increasing weight, increasing education, and increasing median household income (all *P* values < .001) (Table 2). The prevalence of reporting no days of insufficient sleep did not differ by airport noise exposure zone.

When we adjusted for individual characteristics and ZIP Code median household income in multivariate linear and logistic regression models to determine whether controlling for these covariates would affect the relationship between airport exposure noise zone and sleep outcomes, we found no significant differences in outcomes between those who lived in the 3 noise exposure zones and those who lived outside those zones (Table 3).

**Table 3:** Adjusted Odds Ratios^a^ for the Likelihood of Insufficient Sleep or Rest in the Previous 30 Days and β Coefficients^b^ for Days of Insufficient Sleep or Rest Associated With Airport Noise Exposure Zone, Behavioral Risk Factor Surveillance System, 2008 and 2009

Zone	All 30 days,^c^ OR (95% CI)	No Days,^d^ OR (95% CI)	Number of Days, β (95% CI)
≥65 dB	0.86 (0.58 to 1.28)	0.95 (0.69 to 1.32)	0.74 (−1.37 to 1.52)
60 dB to <65 dB	1.04 (0.78 to 1.40)	0.96 (0.77 to 1.21)	0.49 (−0.85 to 1.08)
55 to <60 dB	0.82 (0.66 to 1.00)	0.98 (0.84 to 1.15)	0.32 (−1.02 to 0.25)
Outside exposure zone	1.00 [Reference]	1.00 [Reference]	0.00 [Reference]

Abbreviations: CI, confidence interval; dB, decibel; OR, odds ratio.

a Adjusted ORs and 95% CIs obtained from multivariate logistic regression analyses that included year, age, race/ethnicity, education, income, weight status, and smoking as covariates.

b β coefficients and 95% CIs obtained from multivariate linear regression analyses that included year, age, race/ethnicity, education, income, weight status, and smoking as covariates.

c The likelihood of reporting all 30 days versus zero to 29 days of insufficient sleep or rest in the previous 30 days.

d The likelihood of reporting no days versus 1 or more days of insufficient sleep or rest in the previous 30 days.

## Discussion

In this study, we combined modeled data on airport noise exposure for 95 airports across the United States with individual health outcome data through a GIS spatial overlay operation using the ZIP Code–level geocodes for 745,868 BRFSS respondents. As a result, we were able to infer the relationship between airport noise exposure and self-reported sleep insufficiency at the population level.

This study confirms findings that adults are more likely to have insufficient rest or sleep if they are younger, female, or non-Hispanic black; or have less education (27–29). Furthermore, sleep disorders and sleep loss are associated with individual risk factors such as weight, smoking status, and mental disorders (1,3,4). After we controlled for individual sociodemographic characteristics and ZIP Code-level socioeconomic status, we found no significant associations between airport noise exposure levels and self-reported sleep insufficiency. Our results are consistent with other findings of no association or a weak association of airport noise with sleep disturbance (19–23).

The strength of our study is that the study population included a large sample population and was geographically diverse, including populations from urban, suburban, and rural areas. The BRFSS is the largest population health survey in the United States. No other national health survey has the same coverage or sample size with the same level of geographic information.

The spatial overlay approach developed in this study was used in the FAA Center of Excellence project Aviation-Related Noise Effects on the Elderly (30), in which noise contours for 89 US airports were overlaid with Medicare data for enrollees aged 65 years or older. Medicare data on hospital admissions for cardiovascular disease were evaluated to establish a potential linkage between aviation-related noise and cardiovascular disease.

This study has several limitations. We used ZIP Code centroids and spatial boundaries of noise exposure levels to represent individual exposures. This assumes that all respondents in 1 ZIP Code are exposed to the same noise level. It is likely that noise levels vary within ZIP Codes; noise is an inherently varying phenomenon over space, whereas ZIP Code boundaries are spatially rigid and discrete. The sleep data are self-reported and are a subjective indication of sleep quality and not an objective measurement of sleep duration. More accurate data on sleep can be obtained through actigraphy or polysomnography (31), which are expensive and complicated methods and might not be feasible for national assessments.

The data on the 95 airports included in our data set, although representative of most major airports in the United States, are not exhaustive. We modeled noise exposure in 2008 and 2009 with the INM, where the number of operations was derived from flight path ETMS data and statistical flight track definitions and utilizations. Using data on actual flight trajectories would provide better accuracy for low-level contours, but such information is not readily available and is much harder to process. Additionally, annual average weather conditions are typically used in INM models.

The use of 1 category for all respondents who live outside the 3 airport noise exposure zones of the 95 airports may have introduced bias: some of those respondents may have lived in noise exposure zones near airports not included in our data set. Existing noise exposure data was used to create the 3 noise exposure zones analyzed in this study. Extending the contours to include exposures below DNL 55 dB would have required extensive additional computations, and these were not done. Future studies should consider including lower DNL contours. To assess sensitivity of assignment to a reference group, we conducted 2 additional analyses where the reference group was defined as 1) respondents who lived in ZIP Codes within 5 miles of the DNL 55 dB airport noise-exposure contours and 2) respondents who lived in rural ZIP Codes. In the former, we wished to control for the possibility that respondents living near airports in our study had similar non-airport noise exposures. In the latter, we wished to compare respondents who we assumed had low overall exposures to environmental noise. In both analyses, we used the same 3 specifications of the outcome variable that we used in the main analysis. In neither additional analysis did we find any significant associations between airport noise exposure and self-reported sleep sufficiency, which helps to support the conclusion that our results were not solely attributable to arbitrary assignment of the reference group.

Finally, the measure of exposure — DNL — used in our study reflects both daytime and nighttime operations and represents the highest cumulative level of noise exposure; it may not accurately indicate noise levels experienced by respondents in their residences during typical sleeping hours. Although previous studies showed that most noise metrics are highly correlated, perhaps another metric, such as an equivalent sound level for a 9-hour night (LAEQN) or a sound exposure level (SEL), would be more appropriate for a study of sleep disturbance, especially if such a study were conducted for 1 airport that has a sufficient level of night-time operation. Although the relationship between airport noise exposures and self-reported sleep sufficiency is an important public health issue, other potential downstream health outcomes (high blood pressure, heart disease, and stroke) may warrant further study through the type of analysis conducted in this study.

New or other existing data sets that contain detailed and objective sleep data are needed. Several studies sponsored by the FAA aim to obtain these data. One such study, Research Methods for Understanding Aircraft Noise Annoyance and Sleep Disturbance (32), has 2 objectives: 1) develop and validate a research protocol for a large-scale study of aircraft noise exposure–annoyance response relationships across the United States and 2) propose alternative research methods for field studies to assess the relationship between aircraft noise and sleep disturbance for US airports. The methods developed for this study are used in the FAA Center of Excellence–sponsored project, Design for a US Field Study on the Effect of Aircraft Noise on Sleep (33), to address the feasibility and cost of objective sleep data collection. This analysis led to the suggestion that a combination of actigraphy and a single-channel electrocardiography might be suitable for a large-scale assessment of the effect of aircraft noise on sleep. The data collection protocol developed in these projects is being applied in a residential setting near a US airport.

We assessed a possible correlation between health and aircraft noise for the entire US territory. Ninety-five airports were considered; the large number and the wide geographic distribution of these airports supported the assumption that the results of the analysis are more generalizable than would be the case with data from a limited geographic area. After controlling for individual sociodemographics and ZIP Code–level socioeconomic status, we found no significant associations between airport noise exposure levels and self-reported sleep insufficiency. This research demonstrates feasibility of a US nationwide epidemiological study of the relationship between aircraft noise and sleep by spatially combining existing health survey and environmental exposure data. We also recommend methods for improving study accuracy, some of which are implemented in recent studies sponsored by the FAA.
